# Pneumococcal meningitis in adults in 2014–2018 after introduction of pediatric 13-valent pneumococcal conjugate vaccine in Japan

**DOI:** 10.1038/s41598-022-06950-w

**Published:** 2022-02-23

**Authors:** Bin Chang, Kosuke Tamura, Hiroyuki Fujikura, Hiroshi Watanabe, Yoshinari Tanabe, Koji Kuronuma, Jiro Fujita, Kengo Oshima, Takaya Maruyama, Shuichi Abe, Kei Kasahara, Junichiro Nishi, Tetsuya Kubota, Yuki Kinjo, Yusuke Serizawa, Reiko Shimbashi, Munehisa Fukusumi, Tomoe Shimada, Tomimasa Sunagawa, Motoi Suzuki, Kazunori Oishi, the Adult IPD Study Group, Bin Chang, Kosuke Tamura, Hiroyuki Fujikura, Hiroshi Watanabe, Yoshinari Tanabe, Koji Kuronuma, Jiro Fujita, Kengo Oshima, Takaya Maruyama, Shuichi Abe, Kei Kasahara, Junichiro Nishi, Tetsuya Kubota, Yuki Kinjo, Yusuke Serizawa, Reiko Shimbashi, Munehisa Fukusumi, Tomoe Shimada, Tomimasa Sunagawa, Motoi Suzuki, Kazunori Oishi, Kenji Gotoh, Chikako Tsubata, Hiroki Takahashi, Tetsuji Aoyagi, Masashi Nakamatsu, Naoko Imuta, Akihito Yokoyama, Hiroaki Takeda, Masayuki Ishida

**Affiliations:** 1 Department of Bacteriology I, National Institute of Infectious Diseases, Tokyo, Japan; 2 Toyama Institute of Health, 17-1 Nakataiouyama, Imizu, Toyama 939-0363 Japan; 3 Infectious Disease Surveillance Center, National Institute of Infectious Diseases, Tokyo, Japan; 4 Department of Infection Control and Prevention, Kurume University School of Medicine, Kurume, Japan; 5 Department of Respiratory Medicine, Niigata Prefectural Shibata Hospital, Niigata, Japan; 6 Department of Respiratory Medicine and Allergology, Sapporo Medical University School of Medicine, Hokkaido, Japan; 7 Department of Infectious Diseases, Respiratory and Digestive Medicine, Faculty of Medicine, University of the Ryukyus, Okinawa, Japan; 8 Department of Infectious Diseases, Tohoku University Hospital, Sendai, Miyagi Japan; 9 Mie Prefectural Ichishi Hospital, Tsu, Mie Japan; 10 Yamagata Prefectural Central Hospital, Yamagata, Japan; 11 Center for Infectious Diseases, Nara Medical University, Nara, Japan; 12 Department of Microbiology, Kagoshima University Graduate School of Medical and Dental Sciences, Kagoshima, Japan; 13 Department of Respiratory Medicine and Allergology, Kochi Medical School, Kochi University, Kochi, Japan; 14 Department of Bacteriology, The Jikei University School of Medicine, Tokyo, Japan; 15 Department of Respiratory and Infectious Disease, Niigata Minami Hospital, Niigata, Japan; 16 Department of Respiratory Medicine, Yamagata Saiseikai Hospital, Yamagata, Japan; 17 Chikamori Hospital, Kochi, Japan

**Keywords:** Microbiology, Diseases, Medical research

## Abstract

We assessed the impact of the pediatric 13-valent pneumococcal conjugate vaccine (PCV13) on pneumococcal meningitis in adults in Japan in 2014–2018 by comparing epidemiological characteristics of adults with invasive pneumococcal disease with (n = 222) and without (n = 1258) meningitis. The annual incidence of pneumococcal meningitis in 2016–2018 was 0.20–0.26 cases/100,000 population*.* Age (p < 0.001) and case fatality rate (p = 0.003) were significantly lower in patients with meningitis than in those without meningitis. The odds of developing meningitis were higher in asplenic/hyposplenic or splenectomized patients (adjusted odds ratio [aOR] 2.29, 95% CI 1.27–4.14), for serotypes 10A (aOR 3.26, 95% CI 2.10–5.06) or 23A (aOR 3.91, 95% CI 2.47–6.19), but lower for those aged ≥ 65 years (aOR 0.59, 95% CI 0.44–0.81). PCV13 had an indirect effect on nonmeningitis, but its impact on meningitis was limited because of an increase in non-PCV13 serotypes. Of meningitis isolates, 78 (35.1%) and 3 (1.4%) were penicillin G- or ceftriaxone-resistant, respectively. We also confirmed an association of the *pbp1b*A641C mutation with meningitis (aOR 2.92, 95% CI 1.51–5.65).

## Introduction

*Streptococcus pneumoniae* colonizes the nasopharynx asymptomatically, and often causes pneumococcal disease in children and adults^1,2^. Occasionally, it can enter the bloodstream and cause invasive pneumococcal diseases (IPD) such as meningitis and bacteremia^3,4^. *S. pneumoniae* is the most common cause of bacterial meningitis in children and adults, and causes serious sequelae and death^5–7^. In 2015, there were estimated to be 83,900 cases and 37,900 deaths in children caused by pneumococcal meningitis worldwide^8^. Of all deaths caused by pneumococcal infection, meningitis is estimated to account for 12%. Up to 30% of survivors have some type of neurological or neuro-behavioral sequelae^9^. These include seizures, hearing loss and vision loss, cognitive impairment, neuromotor disability and memory or behavior changes.

*S. pneumoniae* contains five high-molecular-mass penicillin-binding proteins (PBPs), named PBP1a, PBP1b, PBP2x, PBP2a, and PBP2b, as well as the low-molecular-weight PBP3^10^. PBPs 2x, 2b, and 1a play a main role in β-lactam resistance. Mutations in PBP2x and PBP2b result in low levels of such resistance and are prerequisites for high-level β-lactam resistance mediated by an altered PBP1a protein. The relationship between mutations in PBP1b and β-lactam resistance is not yet clear. A recent genome-wide association study from the United States reported a significant association of the *pbp1b*A641C mutation with the clinical occurrence of meningitis in patients with IPD, but there is no evidence that this mutation results in β-lactam resistance^11^. However, it is unclear whether the *pbp1b*A641C mutation is associated with meningitis in adult patients with IPD in Japan.

The incorporation of pneumococcal conjugate vaccines (PCVs) into infant immunization programs worldwide resulted in significant reductions ranging from 41 to 97% in the vaccine-type IPD, including pneumococcal meningitis, in children and older age groups^12^. In Japan, PCV7 was introduced for children under 5 years old in November 2010, and subsequently included in the national immunization program in April 2013. It was then replaced by 13-valent PCV (PCV13) in November 2013, and the vaccination rate of PCV13 in children was approximately 90% in 2014. A significant reduction in the incidence of PCV7-type IPD in children under 5 was reported in Japan after the introduction of PCV7^13^, although the incidence of IPD in children caused by nonvaccine serotypes increased. In 2014, a 23-valent pneumococcal polysaccharide vaccine (PPSV23) was included in the national immunization program for adults aged ≥ 65 years, while PCV13 was licensed for this group in 2014, and became available on a voluntary basis. We conducted an enhanced surveillance of IPD among adults in Japan from 2013 to 2015, and found an indirect effect of the use of the pediatric PCV7 on the epidemiology of adult IPD^14^. Although the epidemiological and bacteriological characteristics of pneumococcal meningitis have been recently reported from Israel, England and Wales^15,16^, the epidemiological features of meningitis have not been fully investigated in adult patients with IPD in Japan.

In this study, we characterized the epidemiological features of pneumococcal meningitis in adults in Japan between 2014 and 2018, and assessed the impact of pediatric PCV13 on this disease.

## Results

### IPD incidence and clinical characteristics of IPD

We enrolled 1480 cases of IPD during the study period. Of these cases, *S. pneumoniae* strain was isolated from a sterile site in 1477 cases, and serotyped with serotype-specific rabbit antiserum^17^. In the remained three cases, the *lytA* gene was detected using blood samples by polymerase chain reaction (PCR) amplification^18^, and the serotype was determined by multiplex serotyping PCR^19^.

The number of IPD cases per 100,000 population in adults gradually increased during 2014–2015, and plateaued during 2016–2018. Therefore, we consider that the number of IPD cases was underreported during 2014–2015, but we were able to estimate that the annual incidence of IPD and pneumococcal meningitis in adults was 1.40–1.98 cases and 0.20–0.26 cases/100,000 population during 2016–2018 (Table 1).

**Table 1:** Annual incidence of pneumococcal meningitis and nonmeningitis cases among adults in Japan during 2016⎼2018.

Year	Incidence by age, cases per 100,000 persons								
	All IPD			Meningitis			Nonmeningitis		
	≥ 15 y	15–64 y	≥ 65 y	≥ 15 y	15–64 y	≥ 65 y	≥ 15 y	15–64 y	≥ 65 y
2016	1.4	0.56	3.14	0.2	0.12	0.35	1.2	0.44	2.79
2017	1.98	0.99	3.97	0.26	0.22	0.34	1.72	0.78	3.63
2018	1.88	0.85	3.9	0.26	0.17	0.46	1.62	0.68	3.45
Mean	1.75	0.8	3.68	0.24	0.17	0.38	1.51	0.63	3.29

*IPD* invasive pneumococcal disease.

A total of 1480 IPD cases were classified as meningitis (n = 222) and nonmeningitis (1258). Of the 222 cases of meningitis, 174 (78.4%) were positive for bacterial culture in cerebrospinal fluid (CSF), 10 (4.5%) were both positive for bacterial culture and pneumococcal antigen in CSF^20^, and 38 (17.1%) were positive in blood cultures and were associated with typical meningeal signs. No case was identified in which the CSF was positive for pneumococcal antigen but negative for bacterial culture. Meningitis cases accounted for 15.0% of the total IPD cases (222/1480). The proportion of meningitis cases to total IPD cases (21.0%; 98/467) in patients aged 15–64 years was significantly higher than in patients ≥ 65 years (12.2%; 124/1013; p < 0.001).

The clinical characteristics of patients with meningitis and nonmeningitis IPD were compared (Table 2). The proportion of men was 59.7%. The median age (range) of patients with meningitis (66 years; age range; 15–100 years) was significantly lower than that of those with nonmeningitis (72 years; age range; 16–103 years; p < 0.001). The case fatality rate (CFR) was significantly lower for meningitis than for nonmeningitis (p = 0.004), although the difference between two groups was not significant when the patients were divided into two age groups. By contrast, the proportion of asplenic/hyposplenic or splenectomized patients was significantly higher for meningitis (9.5%) than for nonmeningitis (3.0%) (p < 0.001). Although the proportion of IPD patients with a history of PCV13 vaccination was only 0.3%, the proportion with a history of PPSV23 vaccination was approximately 10%. No difference was found in the rates of vaccination with PCV13 or PPSV23 between meningitis and nonmeningitis patients.

**Table 2:** Comparison of clinical characteristics between meningitis and nonmeningitis cases among adults in Japan, 2014⎼2018.

Variables	Total IPD (n = 1480)	Meningitis (n = 222)	Nonmeningitis (n = 1258)	p-value by *χ*^2^ test
Male	883 (59.7%)	133 (59.9%)	750 (59.6%)	0.935
**Median age (range)**	71 (15–103)	66 (15–100)	72 (16–103)	< 0.001*
15–64 years	467 (31.6%)	98 (44.1%)	369 (29.3%)	< 0.001
≥ 65 years	1013 (68.4%)	124 (55.9%)	889 (70.7%)	
Smoking history	643 (43.4%)	82 (36.9%)	561 (44.6%)	0.034
Alcohol abuse	257 (17.4%)	43 (19.4%)	214 (17.0%)	0.392
**Immunocompromised conditions**	400 (27.0%)	54 (24.3%)	346 (27.5%)	0.325
Asplenia or hyposplenia or splenectomy	59 (4.1%)	21 (9.5%)	38 (3.0%)	< 0.001
Autoimmune disease	101 (6.8%)	10 (4.5%)	91 (7.2%)	0.137
Corticosteroid therapy	108 (7.3%)	13 (5.9%)	95 (7.6%)	0.370
Solid organ cancer	151 (10.2%)	14 (6.3%)	137 (10.9%)	0.037
Hematologic cancer	60 (4.1%)	5 (2.3%)	55 (4.4%)	0.140
Anti-cancer agent	106 (7.2%)	11 (5.0%)	95 (7.6%)	0.167
**Fatal outcome**	244 (16.5%)	22 (9.9%)	222 (17.6%)	0.004
15–64 years	58/467 (12.4%)	7/98 (7.1%)	51/369 (13.8%)	0.075
≥ 65 years	186/1013 (18.4%)	15/124 (12.1%)	171/889 (19.2%)	0.054
Vaccination history of PCV13	4 (0.3%)	0 (0%)	4 (0.3%)	> 0.999**
Vaccination history of PPSV23	147 (9.9%)	24 (10.8%)	123 (9.8%)	0.635

*Mann–Whitney nonparametric *U* test, **Fisher’s exact test.

### Association of variables with the odds of developing meningitis

We next analyzed variables including age ≥ 65 years, asplenia/hyposplenia or splenectomy, and major serotypes to determine whether they were significantly associated with meningitis after adjusting for confounders (Table 3). The two most common serotypes of meningitis cases were 10A (17.6%) and 23A (16.7%). While the odds of meningitis were higher for asplenic/hyposplenic or splenectomized patients (adjusted odds ratio [aOR] 2.29, 95% CI 1.27–4.14) and for serotypes 10A (aOR 3.26, 95% CI 2.10–5.06) or 23A (aOR 3.91, 95% CI 2.47–6.19), they were lower for those age ≥ 65 years (aOR 0.59, 95% CI 0.44–0.81) or having serotype 19A (aOR 0.20, 95% CI 0.07–0.56).

**Table 3:** Association of serotype with meningitis or nonmeningitis cases among adults in Japan during 2014⎼2018.

Variables	No. (%)			Univariate analysis		Multivariate analysis	
	Total cases	Meningitis	Nonmeningitis	OR (95% CI)	p-value	aOR (95% CI)	p-value
	(n = 1480)	(n = 222)	(n = 1258)				
Age years	71 (62–82)	66 (57–75)	72 (63–83)				
≧65 years	1013 (68.4)	124 (55.9)	889 (70.7)	0.53 (0.39–0.70)	< 0.001	0.59 (0.44–0.81)	< 0.001
Asplenia/ hyposplenia, or splenectomy	59 (4.1%)	21 (9.5%)	38 (3.0%)	3.35 (1.93–5.83)	< 0.001	2.29 (1.27–4.14)	0.006
**Serotype**							
3	187 (12.6)	18 (8.1)	169 (13.4)	0.57 (0.34–0.95)	0.03	0.77 (0.46–1.31)	0.334
12F	154 (10.4)	18 (8.1)	136 (10.8)	0.73 (0.44–1.22)	0.226		
19A	138 (9.3)	4 (1.8)	134 (10.7)	0.15 (0.06–0.42)	< 0.001	0.20 (0.07–0.56)	0.002
10A	112 (7.6)	39 (17.6)	73 (5.8)	3.46 (2.28–5.26)	< 0.001	3.26 (2.10–5.06)	< 0.001
23A	97 (6.6)	37 (16.7)	60 (4.8)	3.99 (2.58–6.19)	< 0.001	3.91 (2.47–6.19)	< 0.001
22F	89 (6.0)	9 (4.1)	80 (6.4)	0.62 (0.31–1.26)	0.187		
35B	81 (5.5)	14 (6.3)	67 (5.3)	1.20 (0.66–2.17)	0.554		
15A	75 (5.1)	14 (6.3)	61 (4.8)	1.32 (0.73–2.41)	0.363		
6C	74 (5.0)	9 (4.1)	65 (5.2)	0.78 (0.38–1.58)	0.484		
11A/E	56 (3.8)	12 (5.4)	44 (3.5)	1.58 (0.82–3.04)	0.173		
Other serotypes	417 (28.2)	48 (21.6)	369 (29.3)				

OR, odds ratio; aOR, adjusted odds ratio; CI, confidence interval. Serotypes responsible for > 50 cases of invasive pneumococcal disease were included for analysis.

### Vaccine coverage

**Figure 1:**
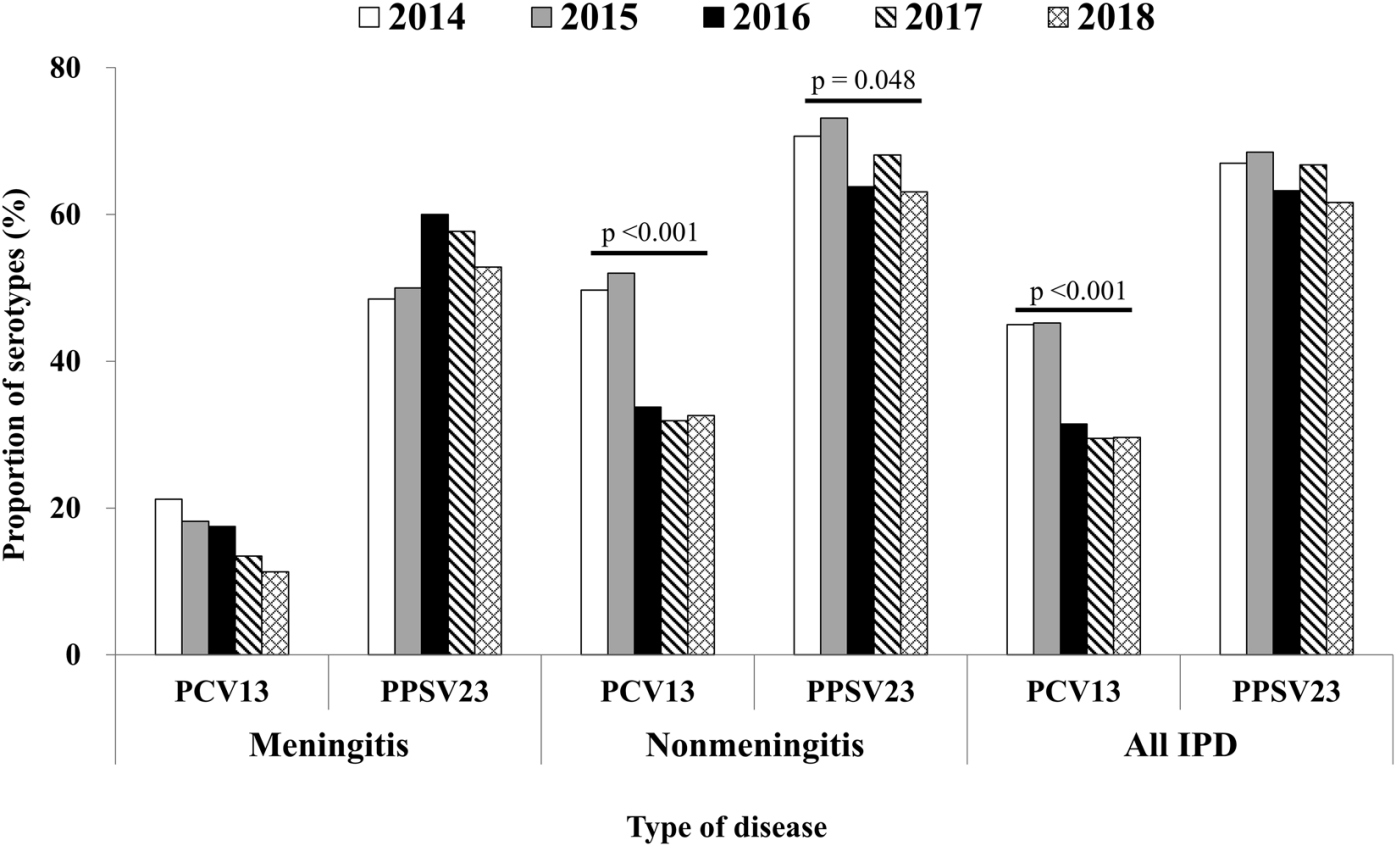
Percentage of vaccine-covered serotypes in pneumococcal isolates from 1480 adult patients with invasive pneumococcal disease in Japan between 2014 and 2018, stratified by year and disease type. PCV13, 13-valent pneumococcal conjugate vaccine; PPSV23, 23-valent pneumococcal polysaccharide vaccine.

The percentages of PCV13 or PPSV23 serotypes within pneumococcal isolates, stratified by isolation year and disease type, are shown in Fig. 1. Over all cases of IPD, the percentages of PCV13 serotypes significantly decreased over the study period (p < 0.001), although no between-year differences were found in the percentage of PPSV23 serotypes. By contrast, no significant between-year differences were found in the percentages of PCV13 serotypes in the meningitis cases, even with the gradual decrease in the percentages of PCV13 serotypes during the study period. The percentages of PPSV23 serotypes in meningitis cases also did not differ over the study period. However, the percentages of PCV13 (p < 0.001) or PPSV23 serotypes (p = 0.048) significantly decreased during the study period in nonmeningitis cases.

### Antimicrobial susceptibility

A total of 1476 strains were examined for antimicrobial susceptibility (Table 4). Of the meningitis cases (n = 222), a total of 78 strains (35.1%) were resistant to penicillin G (PCG). By contrast, of the nonmeningitis isolates (n = 1254), 14 strains (1.1%) showed intermediate resistance (n = 9) or resistance (n = 5) to PCG. All the meningitis strains with serotypes 23A (37 strains) and 15A (14 strains) were resistant to PCG, and strains resistant to PCG were detected for several other serotypes including 35B, and 6C (Appendix Figure). Three meningitis strains were ceftriaxone resistant, but no meropenem-resistant strain was found. All meningitis strains were susceptible to vancomycin. The minimum inhibitory concentration (MIC)_50_ and MIC_90_ values were similar for meningitis and nonmeningitis strains for all antimicrobial agents tested.

**Table 4:** Susceptibility of pneumococcal isolates from meningitis and nonmeningitis cases against four antimicrobial agents.

Antimicrobial agents	The pneumococcal strains isolated from; No of strain (%)									
	Meningitis (n = 222)					Nonmeningitis (n = 1254)				
	Susceptible	Intermediate	Resistant	MIC_50_ (μg/mL)	MIC_90_ (μg/mL)	Susceptible	Intermediate	Resistant	MIC_50_ (μg/mL)	MIC_90_ (μg/mL)
Penicillin G	144 (64.9)	–*	78 (35.1)	0.03	1	1240 (98.9)	9 (0.7)	5 (0.4)	0.03	1
Ceftriaxone	209 (94.1)	10 (4.5)	3 (1.4)	0.25	0.5	1230 (98.1)	12 (1.0)	12 (1.0)	0.25	0.5
Meropenem	208 (93.7)	14 (6.3)	0 (0)	0.015	0.25	1157 (92.3)	87 (6.9)	10 (0.8)	0.015	0.25
Vancomycin	222 (100)	–*	–*	0.25	0.5	1254 (100)	–*	–*	0.25	0.5

* Breakpoint undefined.

### Association of the *pbp1b*A641C mutation with meningitis

We evaluated the effect of the *pbp1b*A641C mutation on the odds of developing meningitis using mixed-effects logistic regression explicitly controlled for patient age group, all pneumococcal serotypes, and susceptibility to three β-lactam antibiotics (Table 5). The odds of causing meningitis were higher for strains bearing the *pbp1b*A641C mutation (aOR 2.92, 95% CI 1.51–5.65), but lower for patients aged ≥ 65 years (aOR 0.55, 95% CI 0.40–0.74). We also evaluated whether the *pbp1b*A641C mutation was associated with the susceptibility to PCG of both meningitis and nonmeningitis isolates (Appendix Table 2). The presence of the *pbp1b*A641C mutation was significantly associated with PCG resistance for meningitis isolates (p < 0.001), but not for nonmeningitis isolates (p = 0.683).

**Table 5:** Logistic regression analysis of isolates from meningitis and nonmeningitis cases in adults on the association between meningitis and *pbp1b*A641C mutation.

Variables	Meningitis (n = 222)		Nonmeningitis (n = 1254)		Univariate logistic regression		Effects	Mixed-effects logistic regression**	
	No	%	No	%	OR (95% CI)	p value		aOR (95% CI)	p value
***pbp1b***A641C** mutation**							Fixed		
No	174	78.4	1152	91.9	Reference			Reference	
Yes	48	21.6	102	8.1	3.12 (2.13–4.55)	< 0.001		2.92 (1.51–5.65)	0.001
**Patient age (years)**							Fixed		
15–64	98	44.1	369	29.4	Reference			Reference	
≥ 65	124	55.9	885	70.6	0.53 (0.39–0.71)	< 0.001		0.55 (0.40–0.74)	< 0.001
Susceptibility to three β-lactam antibiotics*							Random		
Serotypes							Random		

OR, odds ratio; aOR, adjusted odds ratio; CI, confidence interval. *Penicillin G, cefotaxime, and meropenem. **Mixed-effects logistic regression model with binary outcome (meningitis vs nonmeningitis) and the indicated explanatory variables. Calculated by Stata software version 16 (StataCorp LLC, College Station, TX, USA).

## Discussion

In the present study, we determined the annual incidence of pneumococcal meningitis in adults in Japan during the period 2016–2018. The incidence rates of pneumococcal meningitis among patients aged 15–64 years and ≥ 65 years remained unchanged during this period. The incidence of pneumococcal meningitis (0.20 cases/100,000 population) in adults in 2016 was approximately one fourth of that (0.85) reported in Israel in 2014–2015, and similar to that (0.29) in England and Wales in 2015–2016^15,16^. We also found that the CFR of adult patients with IPD was significantly lower for those with meningitis (9.9%) than for nonmeningitis cases (17.6%), consistent with a report from Israel^15^. The lower CFR of adult patients with meningitis in our study might be explained partially by the significantly lower age of adult patients with meningitis compared with those with nonmeningitis.

Our study also demonstrated that the proportion of asplenic/hyposplenic or splenectomized adult patients with meningitis (9.5%) was significantly higher than for those with nonmeningitis (3.0%), as was the aOR for meningitis. Collectively, our data indicate that impaired splenic function may increase the odds of meningitis irrespective of the infecting serotype or the patient’s age. It is well known that asplenic or hyposplenic or splenectomized patients are at increased risk for fulminant infections with encapsulated bacteria; this is attributable to a lack of splenic filtering and decreased production of specific antibodies and memory B cells^21,22^. A recent study of 2435 adult patients with IPD demonstrated that the proportion of asplenic patients with meningitis (6/37; 21.0%) was significantly higher than in patients with a spleen (112/2398; 4.7%)^23^. The authors also reported that the proportions of asplenic patients requiring intensive care admission or mechanical ventilation use and suffering acute kidney injury were significantly higher than for patients with a spleen, although the difference in the CFR between the two groups was not significant. These findings confirmed that asplenic patients had more severe IPD than patients with a spleen.

We also found significantly higher odds of meningitis in patients infected with serotypes 10A or 23A, which are not included in PCV13. A recent study in England and Wales reported similar findings of significantly increased odds of meningitis with serotypes 10A, 23B, and 35B^16^. Another study from Israel also demonstrated that the percentage of adult patients with meningitis was significantly higher for IPD caused by serotypes 24F, 23F, 15B/C, 23B, or 23A^15^. These findings indicate that the serotypes that commonly cause meningitis in adults include both types contained in PPSV23 (such as 10A and 15B/C) and nonvaccine types (such as 23A, 23B, 24F), plus 23F, and support the idea that there is limited impact of pediatric PCV13 on meningitis in adults. A recent study from the Pneumococcal Serotype Replacement and Distribution Estimation project assessed the serotype distribution of the remaining serotypes involved in pneumococcal meningitis worldwide^24^. The study demonstrated the percentage of pneumococcal meningitis occurring after infection with serotypes included in the current PCV13 and upcoming PCV products including PCV20 or PCV24, for all cases of meningitis in locations using PCV13^25–28^. While the percentage of PCV13 serotypes was 14.8% for patients aged < 5 years and 25.2% for those aged ≥ 5 years, the percentages of PCV20 or PCV24 serotypes were 56.5%*–*57.3% and 61.4*–*63.4%, respectively, for patients of all ages^24^. The higher percentages of PCV20 or PCV24 serotypes in cases of meningitis indicate that the higher-valency PCVs have enhanced potential to prevent more cases of pneumococcal meningitis in children and adults.

Because the rate of vaccination with PCV13 in Japanese adults is currently negligible, the significant decrease of the percentage of PCV13 serotypes in all cases of IPD during the study period suggests an indirect effect of pediatric PCV13 vaccination. Although no difference was found in the percentage of PPSV23 serotypes for total cases of IPD, a slight, but significant decrease was found in the percentage of PPSV23 serotypes in nonmeningitis cases, but not in meningitis cases. This may be because the indirect effect of PCV13 is more evident in nonmeningitis than in meningitis: although the percentage of PCV13 serotypes was significantly decreased in nonmeningitis cases, no significant difference was found in meningitis cases. This finding indicates that the indirect effect of pediatric PCV13 vaccination on meningitis in adults in Japan has a limited impact, probably because of an increase in cases caused by the non-PCV13 serotypes, such as 10A or 23A. We also noted that there was a small, but consistent decease from 2014 to 2018 in meningitis cases. This could be attributable to our inability to detect a statistical decrease because of the relatively small number of cases caused by PCV13 serotypes. A study from Israel also reported that nonmeningitis IPD but not meningitis decreased after PCV13 implementation^15^. The authors suggested that this was because of an increase in cases caused by non-PCV13 serotypes. By contrast, a study in England and Wales reported that the replacement of PCV7 by PCV13 in 2010 decreased the incidence of pneumococcal meningitis, mainly those caused by the additional serotypes included in PCV13, without any increase in cases caused by non-PCV13 serotypes^16^.

In our study, 35.1% of pneumococcal isolates (n = 222) from patients with meningitis were PCG resistant. By contrast, only 0.7% and 0.4% of pneumococcal isolates (n = 1254) from nonmeningitis cases showed intermediate resistance or resistance to PCG. The difference in the MIC values for PCG between meningitis and nonmeningitis cases was responsible for the different MIC breakpoints for PCG, because the values of MIC_50_ and MIC_90_ for each antimicrobial agent were similar for the two groups. Based on this finding of reduced β-lactam susceptibility, it may be advisable for clinicians to administer ceftriaxone or meropenem plus vancomycin for adult patients suspected of having pneumococcal meningitis until susceptibility results are reported^6,29^. Japanese investigators recently reported that all patients with pneumococcal meningitis were treated with two or more antibiotics^30^. In that study, antimicrobial treatment was frequently initiated with ceftriaxone, followed by sulbactam/ampicillin, tazobactam/piperacillin, and vancomycin.

Here we found that the odds of meningitis were higher in the presence of the *pbp1b*A641C mutation (aOR 2.92, 95% CI 1.51–5.65), and lower for patients aged ≥ 65 years (aOR 0.55, 95% CI 0.40–0.74), although we also found a significant association between the *pbp1b*A641C mutation and PCG resistance. These data confirm the previously reported association of the *pbp1b*A641C mutation with meningitis^11^.

This study had several limitations. First, 33.1% of all 2213 cases reported to the National Epidemiological Surveillance of Infectious Disease from 10 prefectures in Japan during the study period were not enrolled in this study. Second, the reporting of some variables was incomplete for some enrolled cases. Therefore, the results of our study may not be fully representative of the population of interest. Third, abdominal computed tomography scans were not examined for all enrolled cases to detect asplenia or hyposplenia. The clinical information about splenectomy may be inadequate. Therefore, we might have underestimated the number of IPD patients with asplenia/hyposplenia or splenectomy.

In conclusion, the incidence of pneumococcal meningitis in adults remained unchanged during 2016–2018. Patient ages and the CFR were significantly lower in meningitis cases than in nonmeningitis cases. The odds of developing meningitis were higher for asplenic/hyposplenic or splenectomized patients and for infection with serotypes 10A or 23A. An indirect effect of pediatric PCV13 on nonmeningitis cases in adults in Japan was evident, but its impact on meningitis cases was limited because of an increase in cases caused by non-PCV13 serotypes.

## Materials and methods

### IPD surveillance, case definition, bacterial strains and serotyping

The Adult IPD Study Group (https://www.niid.go.jp/niid/ja/ibi.html) conducted population-based surveillance of IPD in Japan from January 2014 to December 2018. IPD occurring in people over the age of 15 who lived in 10 prefectures (Hokkaido, Miyagi, Yamagata, Niigata, Mie, Nara, Kochi, Fukuoka, Kagoshima, and Okinawa) was included in this surveillance. A case of IPD was defined as isolation of *S. pneumoniae* by bacterial culture or detection of *S. pneumoniae*-specific DNA targeting *lytA* by PCR amplification^18^ from a sterile site such as blood or CSF, or a positive test result of pneumococcal antigen in CSF. As the routine procedure, we isolated *S. pneumoniae* in samples from the sterile sites, and pneumococcal isolate was serotyped by a capsule Quellung reaction with serotype-specific rabbit antiserum (Statens Serum Institute, Copenhagen, Denmark), as described^17^. Detection of *S. pneumoniae*-specific DNA targeting *lytA* and the serotyping by multiplex serotyping PCR for the cases with a positive reaction from the initial PCR were carried out in response to the request from clinicians at the Department of Bacteriology I, National Institute of Infectious Diseases (NIID), although these were not done routinely. Pneumococcal antigen testing in CSF was performed as a supportive diagnosis for pneumococcal meningitis at a hospital laboratory.

Pneumococcal isolates were identified by commonly used methods such as colony morphology on sheep blood agar, etc., alpha-hemolysis, optochin-sensitivity, and bile solubility at a hospital laboratory^19^. As a general procedure, the pneumococcal strain isolated on 5% sheep blood agar, etc. at a hospital laboratory was collected by the public health center, and sent to the prefectural public health institute. The prefectural public health institute sent the isolate in transport medium to NIID by a special parcel delivery. When the bacterial isolate showed positive reaction with pneumococcal group antiserum 11 and factor antiserum 11c, but showed negative reaction with factor antisera 11b, 11f, and 11g using the Quellung reaction, the serotype of the strain was determined to be type 11A/E. When the bacterial isolate did not show a positive reaction with any antiserum, and no obvious capsule was detected by India ink staining, the serotype was determined to be nontypeable^31^.

All IPD cases were classified into two groups: meningitis or nonmeningitis. A meningitis case was defined as IPD with typical meningeal signs, and nonmeningitis as IPD other than meningitis. Pneumococcal meningitis in adults often appears rapidly with typical symptoms such as high fever, headache, altered mental status and neck stiffness^32^. The annual incidence rates of IPD and pneumococcal meningitis were calculated based on the population denominators of the 10 prefectures obtained from the Statistics Bureau of Japan^33^. When a case of IPD was reported to the Study Group, clinical information and the pneumococcal isolates were sent to NIID. The clinical information included sex, age, smoking history, alcohol use, history of pneumococcal vaccination, current underlying diseases, immunocompromised conditions, asplenia/splenic hypoplasia (hyposplenia) or splenectomy, and fatal outcome. One isolate per case was included. Neither the laboratory methods nor the procedures of specimen collection have changed during the study period.

### Antimicrobial susceptibility test

Because one of the isolates did not grow under the conditions used for the antimicrobial susceptibility test, 1476 strains were examined by the microbroth dilution method according to the Clinical and Laboratory Standards Institute (CLSI) guidelines*.* The MIC breakpoints were determined for PCG, ceftriaxone, meropenem, and vancomycin according to the CLSI criteria^34^. These antimicrobial agents were included in antimicrobial susceptibility tests, because β-lactam antibiotics are commonly used to treat pneumococcal infections and vancomycin is the first-line drug for treatment of meningitis caused by penicillin-resistant *S. pneumoniae*. In addition, the CLSI has stated that penicillin, ceftriaxone, or meropenem should be tested by a reliable MIC method and reported routinely for pneumococcal isolates from CSF^34^. Such isolates can also be tested against vancomycin using the MIC or disk diffusion method.

### Detection of a point mutation in the *pbp1b* gene

Primer sequences, PCR amplification, and sequencing for detection of the *pbp1b*A641C mutation in 1477 pneumococcal strains were performed as previously described ^11^.

### Statistical analysis

The demographic data and clinical characteristics of patients with meningitis and nonmeningitis IPD were compared using the *χ*^2^ test, Fisher’s exact test, or the Mann–Whitney nonparametric *U* test. The odds for serotypes of strains of developing meningitis were estimated using multivariate logistic regression model, adjusted by the variables higher for meningitis cases in univariate analysis. Categorical variables are presented as the number and frequency, and continuous variables as means (± standard deviations) or median (range). The Mantel–Haenszel test for trend was used to evaluate the trend in vaccine coverage from 2014 to 2018. The effect of the *pbp1b*A641C mutation on meningitis was estimated using a mixed-effects logistic regression model as described^11^. All statistical analyses were performed using IBM SPSS Statistics version 24 (IBM Corp., Armonk, NY, USA) or Stata software version 16 (StataCorp LLC, College Station, TX, USA). Statistical significance was set at p < 0.05.

### Ethics statement

This study was reviewed and approved by the Medical Research Ethics Committee of National Institute of Infectious Diseases for the Use of Medical Subjects (approval no. 707), and was conducted according to the principles expressed in the Declaration of Helsinki. The requirement for informed consent was waived because the data do not contain any patient identifiers and samples were taken as part of standard patient care.

## Supplementary Information

Supplementary Information.

## Abbreviations

IPD: Invasive pneumococcal disease
PBPs: Penicillin-binding proteins
PCV: Pneumococcal conjugate vaccine
PPSV23: 23-Valent pneumococcal polysaccharide vaccine
PCR: Polymerase chain reaction
CFR: Case fatality rate
PCG: Penicillin G
MIC: Minimum inhibitory concentration
CSF: Cerebrospinal fluid
CLSI: Clinical and Laboratory Standards Institute
NIID: National Institute of Infectious Diseases
