# Supplementary material for: Pneumococcal meningitis in adults in 2014–2018 after introduction of pediatric 13-valent pneumococcal conjugate vaccine in Japan
Source: Sci Rep. 2022 Feb 23;12:3066. doi: 10.1038/s41598-022-06950-w (PMC8866494; doi:10.1038/s41598-022-06950-w)
Supplement: Supplementary file 1 — Supplementary Information. [file 41598_2022_6950_MOESM1_ESM.pdf]

Title: Pneumococcal Meningitis in Adults in 2014–2018 after Introduction of Pediatric 13-valent Pneumococcal Conjugate Vaccine in Japan

Authors:

Bin Chang, Kosuke Tamura, Hiroyuki Fujikura, Hiroshi Watanabe, Yoshinari Tanabe, Koji Kuronuma, Jiro Fujita, Kengo Oshima, Takaya Maruyama, Shuichi Abe, Kei Kasahara, Junichiro Nishi, Tetsuya Kubota, Yuki Kinjo, Yusuke Serizawa, Reiko Shimbashi, Munehisa Fukusumi, Tomimasa Sunagawa, Motoi Suzuki, Kazunori Oishi, and the Adult IPD Study Group

Supplementary Table 1. Distribution of *pbp1bA641C* mutation in isolates from meningitis and nonmeningitis cases in adults by serotype.

Serotypes	No. of strains with mutation	Meningitis		Nonmeningitis	
		Mutation (+)	Mutation (-)	Mutation (+)	Mutation (-)
23A	97	36	0	60	1
6C	74	6	3	22	43
6A	24	1	2	7	14
16F	11	0	1	4	6
23B	10	3	0	1	6
23F	19	0	1	3	15
6B	38	1	3	1	33
3	186	0	18	2	166
14	23	0	0	1	22
15B	26	0	4	1	21
NT	4	1	0	0	3
In total	150	48	32	102	330

NT: nontypeable.

Supplementary Table 2. Relationship between *pbp1bA641C* mutation and susceptibility of penicillin G in isolates from meningitis and nonmeningitis cases in adults.

Susceptibility (Breakpoint)	With *pbp1bA641C* mutation		Without *pbp1bA641C* mutation		*p* value
Meningitis isolates (n=222)	No.	%	No.	%	
MIC for PCG ≤ 0.06	5	10.4	139	79.9	<0.001
MIC for PCG ≥ 0.12	43	89.6	35	20.1	
Nonmeningitis isolates (n=1254)	No.	%	No.	%	
MIC for PCG ≤ 2	101	99.0	1,139	98.9	0.683
MIC for PCG ≥ 4	1	1.0	13	1.1	

PCG: penicillin G.

Figure S1. Number of penicillin G-sensitive and -resistant pneumococcal isolates from adult patients with meningitis stratified by serotype.

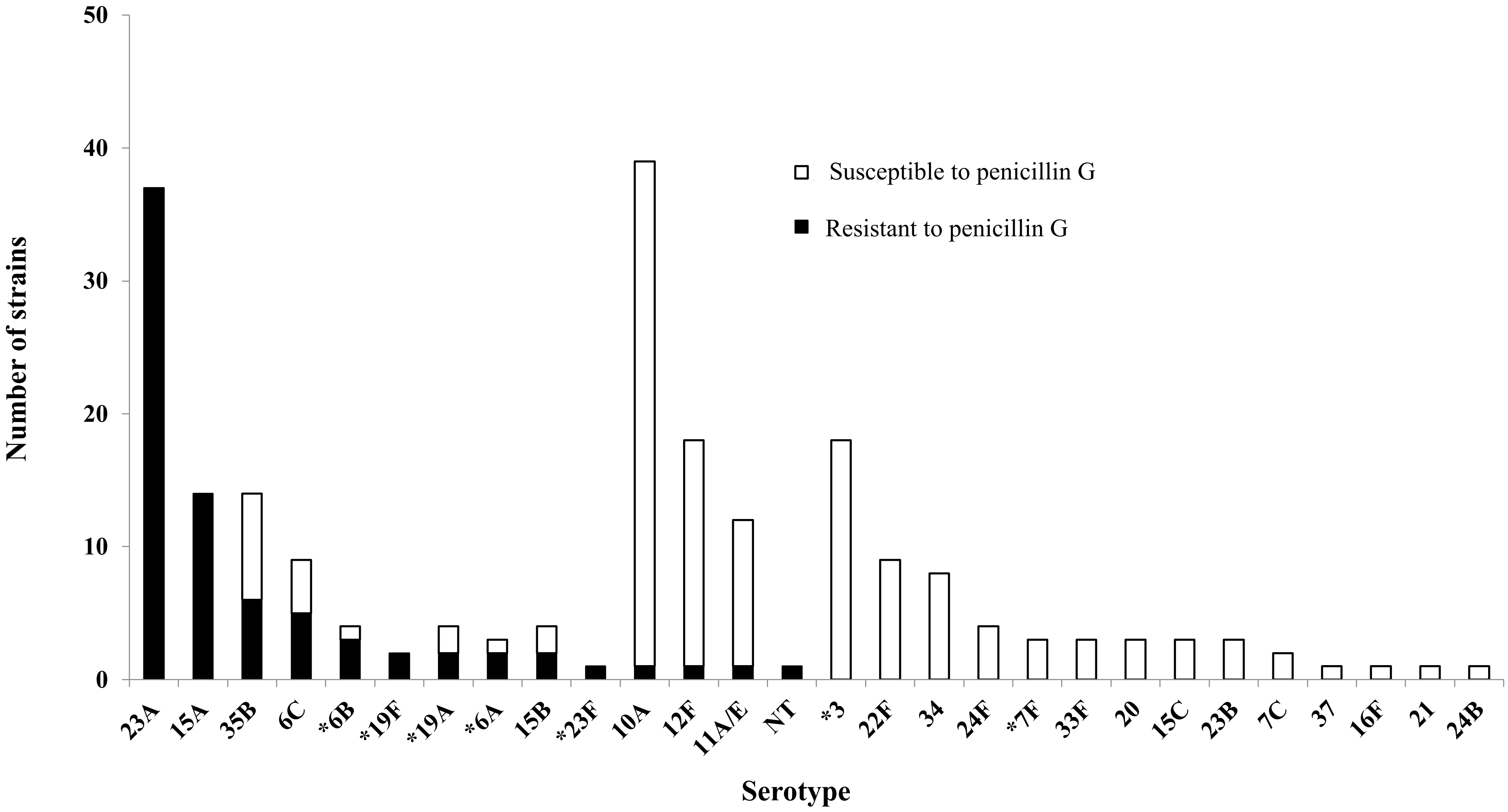

NT, nontypeable. *Serotypes contained in 13-valent pneumococcal conjugate vaccine.

The Adult IPD Study Group

Michiharu Nakano[1], Makoto Ishiguro[2], Takuya Ojima[2], Junji Seto[3], Yuki Kimura[4], Mikako Hosoya[4], Satoru Yamazaki[5], Yuhki Nagai[6], Akihito Kobayashi[6], Yoshie Uchida[7], Sayaka Osaki[8], Atsuko Kageyama[8], Yuki Carle[9], Akira Ohishi[9], Yoshiki Etoh[9], Hiroaki Shigemura[9], Naomi Shinkawa[10], Tetsuya Kakita[11]

[1]Hokkaido Institute of Public Health
[2]Sapporo City Institute of Public Health
[3]Yamagata Prefectural Institute of Public Health;
[4] Niigata Prefectural Institute of Public Health and Environmental Sciences
[5]Health and Sanitation Department of Niigata City
[6]Mie Prefecture Health and Environment Research Institute
[7]Nara Prefectural Institute of Health
[8]The Public Health Institute of Kochi Prefecture
[9]Fukuoka Institute of Health and Environmental Science
[10]Center for Environment and Health, Kagoshima Prefecture
[11]Okinawa Prefectural Institute of Health and Environment
